# *Croatian Medical Journal* – an encomium: *vivat, crescat, floreat*!

**DOI:** 10.3325/cmj.2022.63.410

**Published:** 2022-10

**Authors:** Ivan Damjanov

**Affiliations:** Emeritus Professor of Pathology, The University of Kansas School of Medicine

As a retired pathologist, I would like to start this text with a pathology-related story about Japan from more than 100 years ago. I read it as a novice pathology trainee and it remained in my memory for many, many years. I hope that you will understand why I decided to retell it here.

At the end of the nineteenth and the beginning of the twentieth century, Japan opened up to the West, trying to modernize and catch up with the rest of the world. These efforts also included establishing professional contacts with European medicine, and especially with German physicians. One of the Germans invited to visit Japan was Ludwig Aschoff (1866-1942), Chairman of the Institute of Pathology in Freiburg im Breisgau. He accepted the invitation and traveled for a few weeks by boat to Japan. During his visit, he delivered a few lectures, inspected the medical facilities, and made recommendations on how to improve the pathology services. At the end of the visit, he wrote into the visitors’ book the following words in German: “*Becharrlichkeit führt zum Ziel*!” (in English translation “Persistence leads to the goal!”).

Several years later, Aschoff was reinvited to see how much Japanese pathology improved. He was most favorably impressed. Under his original exhortation, he added another sentence: “*Becharrlichkeit hat zum Ziel geführt*!” (in English translation “ With persistence the goal was reached!”). In this context, it is worth a mention that Japanese have since then caught up with the Western World, indeed, and received 28 Nobel Prizes, five of which were in physiology and medicine.

The message of this story about persistence may be applied to the editors, members of editorial board, and the Deans of the four Croatian Medical schools who became the founding fathers of the *Croatian Medical Journal* (*CMJ*). Obviously, all this would not have been possible without the wide support of Croatian physicians and scientists, and all the others who have donated their free time to publishing the *CMJ* over the last 30 years. Special credit goes to the Founding Editors, who made the *CMJ* happen by conceiving the idea of a high-quality medical journal that coincided with the creation of the new state of Croatia; by realizing the need for such a journal in our country; and by translating their vision into printed pages. Without their vision, dedication, and persistence, the *CMJ* would not have become what it is today.

In comparison with many others, my own contribution to this joint effort was miniscule, as I served as the Co-editor in Chief of the *CMJ* for only a very short period of time. Still, my short stint in that position helped maintain the continuity in the life of the Journal and facilitated the transition of the Editorship from one generation to another. Continuity is very important in medical journal publishing, and thus, I am glad that I was invited to give my part to this end. In that short period of time I learned a lot. I was also given the opportunity to see first-hand some of the problems that previous editors experienced and think of some that the future ones might anticipate. I also saw how well organized the entire editorial operation was. I felt reassured that editorial team would function even without my input and continue to do so flawlessly for many years to come. The fact that we are here for 30 years since the day the journal was launched shows that I was correct in my assessment.

I like quotes, and thus allow me to finish with two quotes that guided me through my life. The first is a quote by the British statesman and writer Benjamin Disraeli. It is a variation on the theme mentioned in the anecdote about Japan and it goes as follows: “The secret of success is consistency of purpose.” If you have a purpose and transmit the belief in your purpose to others the success will follow.

The second quote is from the French writer André Gide: ”*Le problème n’est pas comment reussir mais comment durer*” (in English translation: “The problem is not how to succeed but how to last”). Since the *CMJ* has survived already 30 years, one can rightfully predict that it will last for much, much longer. May it live, grow, and flourish, or as one would say it in Latin: *Vivat, crescat, floreat*!

